# The Trojan Chicken Study, Minnesota

**DOI:** 10.3201/eid1205.050790

**Published:** 2006-05

**Authors:** Sandra R. Olson, Gregory C. Gray

**Affiliations:** * University of Iowa College of Public Health, Iowa City, Iowa, USA;; † University of Wisconsin-Madison, Madison, Wisconsin, USA

**Keywords:** Influenza, respiratory tract infections, epidemiology, poultry, disease outbreaks, disease transmission, environmental factors, public health, research

## Abstract

We conducted a study in the summer of 2004 at county fairs in the Midwest to investigate the role poultry exhibits have in spreading avian pathogens to humans. A nearly invisible powder (pathogen surrogate) that fluoresces under UV light was surreptitiously sprinkled each day on 1 show bird at each of 2 fairs. A UV light box was used to daily examine the hands of 94 poultry-exhibit participants (blinded regarding UV box results) for up to 4 days during the poultry shows. Enrollment and end-of-study questionnaires collected data on pathogen risk factors. Eight (8.5%) of 94 participants had evidence of fluorescent powder contamination (95% confidence interval 2.76%–14.26%). This contamination and infrequent handwashing practices suggest that county fairs are a possible venue for animal-to-human pathogen transmission.

## Abstract 2

County fairs are a possible venue for animal-to-human pathogen transmission.

Recently, the Centers for Disease Control and Prevention (CDC) declared avian influenza to be the world's number-1 health threat (*1*); in particular, the wide and rapid spread of the H5N1 strain has heightened concerns. All H5N1 cases to date have been associated with direct contact with poultry, but recently, human-to-human transmission has been purported in Thailand (*2*). Previously healthy children and young adults seem to be especially susceptible to this illness (*3*). As of February 27, 2006, a total of 173 confirmed human cases of avian influenza A (H5N1) and 93 deaths have been reported to the World Health Organization, for a case-fatality rate of 53.8% (*4*).

Close contact with live poultry has been implicated in recent outbreaks of avian influenza in humans in Southeast Asia and elsewhere (*2**,**5**–**8*). In the 1997 Hong Kong outbreak, live bird markets were implicated as the source of exposure to the virus (*8*). In the United States, live bird markets are a known reservoir for avian influenza (*9**–**11*), but thus far they have not been associated with human avian influenza infection. Live bird markets involve a mixing of birds from diverse areas, crowded conditions for humans and livestock, mixing of different species of animal, and often a lack of proper sanitation, thus providing opportunity for outbreaks of disease. Transport of animals to market is a source of stress than can induce increased shedding of infectious agents. Stressed birds are also more susceptible to infections (*12*).

While live bird markets are uncommon in the Midwest, animal exhibits such as those at county fairs are quite common. Such exhibits are similar to live bird markets in that they involve transport and mixing of animals from different locations, crowded conditions, and a general lack of sanitation. Approximately 125 million people visit agricultural fairs every year in the United States (*13*). Fairs usually involve close proximity of food vendors to animal exhibits. Many animal exhibits encourage or allow visitors to touch animals. Small children are frequent visitors to county fairs and animal exhibits, and children also engage in behavior such as nail biting that may make them more likely to ingest infectious agents. Live animal exhibits such as petting zoos and open farms, which are in many ways similar to county fairs, have also been implicated in outbreaks of *Escherichia coli* O157:H7 and other bacterial diseases (*13**,**14*).

Proper handwashing is recommended to protect persons from infection (*15*). However, animal exhibits often lack adequate handwashing facilities, and many persons may be unaware of the risk such exhibits pose. Direct contact with animals, indirect contact with contaminated objects, or inhalation of aerosolized virus could contribute to transmission of pathogens in such settings.

Because little is known about the possible spread of pathogens at county fairs, and because most cases of avian influenza have resulted from close contact with poultry, a study was undertaken to model interspecies transmission of pathogens at county fair poultry shows. The specific aims of this study were to determine the proportion of human poultry show participants who demonstrate hand contamination by a surrogate marker for an avian pathogen and to determine possible risk factors associated with such contamination.

## Materials and Methods

A feasibility study was conducted at a county fair in Iowa (county A) to evaluate study methods. After the feasibility study, human poultry fair participants were enrolled at a larger county fair in Minnesota (county B). Both fairs were held within small cities with populations of ≈100,000.

At county fairs, poultry judging often takes place in show areas that are open to the public. Birds are usually placed in cages that are stacked one upon another and set upon tables (Figure). Because poultry classes are judged separately and competitors may show their birds in several poultry classes, birds are frequently moved in and out of their cages for grooming and competition. During the competition, birds are moved to competition cages that have previously housed birds from other competition classes. Judges typically handle each bird individually; they take the bird from the exhibitor, examine it, and then hand it back to the exhibitor (Figure). Handwashing is not generally performed as the judge moves from bird to bird, nor is handwashing common before or after exhibitors handle their birds. After competition, birds often remain on exhibit for several days, and they may be touched by the general public.

**Figure:**
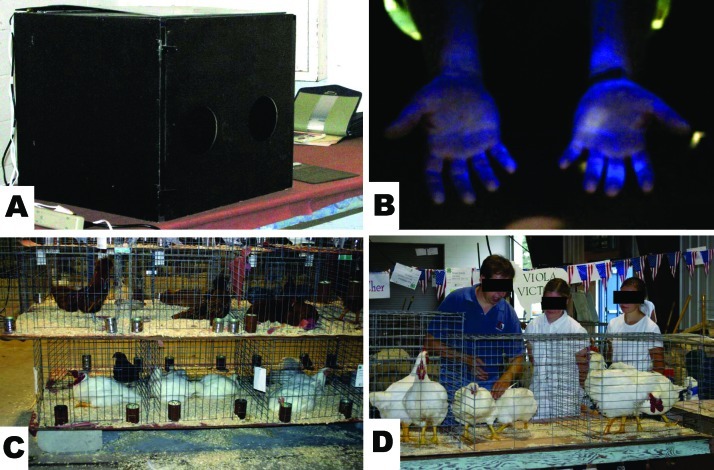
A) UV light box for screening hands for evidence of contamination with fluorescent dye; B) example of fluorescence on contaminated hands; C) stacked poultry in cages at a county fair; D) poultry judge moved from cage to cage, handling each bird and passing bird to exhibitor.

This study was reviewed and approved by the University of Iowa's Institutional Review Board and Animal Use and Care Committee. The investigators participated in online human and animal subjects training. Informed consent was sought from participants before they were enrolled.

Anyone >7 years of age present in the poultry exhibit area at any time during the period when poultry were on active exhibit was eligible to enroll in the study. Recruitment focused on members of 4-H clubs and open-class exhibitors, their families, and 4-H club staff, but also included other visitors. Enrollment occurred continuously over a 4-day period (Monday through Thursday) while poultry were exhibited at the fairs. A special sign and an information table were used to promote the study. Study participants were recruited for enrollment as they walked through the poultry exhibit area. After providing informed consent, study participants were asked to complete a 1-page questionnaire that gathered demographic and poultry exposure data. Participants were also asked to complete a 1-page end-of-study questionnaire after they completed their experience at the poultry exhibit (day 4). This instrument gathered data on handwashing and types of animals handled at the fair.

GloGerm (GloGerm Company, Moab, UT, USA), a benign, synthetic, organic colorant A-594-5 that fluoresces under a black light, was used as a surrogate marker for an avian pathogen. This powder (also found in liquid or gel form) is commonly used in handwashing training in hospitals and businesses (*16*). Each day, the white powder was surreptitiously applied to the same single chicken at the fair to imitate a single source of pathogen. White broiler chickens were chosen as the exposure birds since the powder was not detectable on their feathers. Each "Trojan chicken" was otherwise treated the same as the other chickens in the poultry shows. While county fair authorities gave permission for the study, neither the judge nor the study participants were aware of neither the surrogate exposure nor which of the chickens were of particular hygienic concern. Instead several participants remarked that they thought the UV light box (see below) in which photographs were taken could somehow detect generic bacterial contamination on the hands.

At county A, chicken powdering was conducted early in the mornings of the 3 days of competition, when competitors were not at the poultry exhibit. At county B, the same strategy was followed but the Trojan chicken was also surreptitiously powdered again in the early afternoon for 3 days of the show. During the powdering, approximately one-third cup powder was liberally sprinkled onto the underside of the chicken to imitate fecal shedding of pathogen. The chicken was then returned to its cage. The Trojan chickens each shared their cage with another, very similar, white broiler chicken, since these birds are normally shown in matched pairs.

To evaluate potential avian influenza transmission, a 2 × 2 × 2-foot wooden box was constructed from plywood. Three black 1-foot × 18-inch fluorescent lights (15 watts) and 1 white 1-foot × 18-inch fluorescent light (15 watts) were mounted under the lid of this isolation box. Study participants inserted their hands through hand holes in 1 side, and they were blinded as to the result of the fluorescence examination of their hands. From an opening in the box on the opposite site, digital photographs of the ventral and dorsal images of the hands were taken with a digital camera (Figure). A log was kept to match the sequentially captured photograph numbers with the participants' names (data were later de-identified). Beginning on day 1 of each poultry show, daily photographs were taken of study participants' hands under the black lights (Figure). Photographs continued to be taken through the afternoon of day 4 (last day of the shows).

Statistical analysis was performed with SAS version 8.0 (Cary, NC, USA). Chi-squared analysis and Fisher exact test were used to compare categorical variables with powder contamination. We used *t* tests to compare continuous variables. Logistic regression modeling was attempted, but the models did not converge. Odds ratios and confidence intervals were calculated by using EpiInfo (CDC, Atlanta, GA, USA) (Table 1).

**Table 1:** Hand contamination by variables gender, age, and roles*

Variable	Not contaminated, n = 86 (%)	Contaminated, n = 8 (%)	OR (95% CI)
Sex			
Female	60 (92.3)	5 (7.7)	Referent
Male	26 (89.7)	3 (10.3)	1.4 (0.2–7.7)
Age group, y			
7–12	12 (92.3)	1 (7.7)	0.3 (0.0–3.3)
13–21	18 (100)	0 (0)	Referent
22–50	33 (89.2)	4 (10.8)	0.4 (0.1–2.3)
51–79	12 (85.7)	2 (14.3)	0.6 (0.1–4.5)
Role			
Exhibitor	18 (100)	0 (0)	Referent
Family member of exhibitor	14 (82.4)	3 (17.6)	0.8 (0.1–4.8)
Visitor	35 (92.1)	3 (7.9)	0.3 (0–1.8)
Other	8 (88.9)	1 (11.1)	0.5 (0–5.2)

*OR, odds ratio; CI, confidence interval. All OR were calculated with exact CI by EpiInfo version 3.3.2 (CDC, Atlanta, GA, USA). The value 0.5 was inserted into cells with values of zero.

## Results

Ninety-four persons participated in the study by having their hands photographed. Among these were 30 poultry exhibitors (Table 2). Of the study participants, 82 (87.2%) completed the enrollment questionnaire, and 44 (46.8%) completed the end-of-study questionnaire. Of all participants in county B, 29 (30.9%) were male.

**Table 2:** Characteristics of participants (N = 94)

Characteristic	n (%)
Completed questionnaire 1	82 (87.2)
Completed questionnaire 2	44 (46.8)
Sex	
Male	29 (30.9)
Female	65 (69.1)
Farm resident	63 (67.0)
Role	
Exhibitor	18 (22.0)
Family member of exhibitor	17 (20.7)
Visitor	38 (46.3)
Other	9 (11.0)
Age, y	
Mean 33	
SD 17.9	
Range 7–79	

The mean age of those who completed the enrollment questionnaire was 33 (range 7–79) years. Eighteen participants were poultry exhibitors, who showed 1–10 birds each (mean 3.4). Fifty-five participants (67.1%) of 82 were residents of farms.

Eight participants exhibited hand contamination (Table 1). Of these, all 8 completed the enrollment questionnaire, and 7 completed the end-of-study questionnaire. Participant gender and hand contamination were not associated. Of participants whose hands were contaminated, 3 were male and 5 were female. None of the persons whose hands were contaminated were exhibitors: 3 were family members of exhibitors, 3 were visitors, and 1 was in the "other" category.

In the age group of 7 to 12 years, 1 (7.7%) participant had hand contamination (Table 1). None of the participants in the 13- to 21-year age group showed hand contamination. Four participants (10.8%) in the 22- to 50-year age group had contaminated hands, and 2 participants (14.3%) who were >51 years showed hand contamination. Contamination rates did not differ by age group.

## Discussion

Our study demonstrated that pathogen transmission is possible through poultry handling at county fairs. A contact transmission proportion of 8.5% (8 persons of the 94 participants had contaminated hands) is high, when one considers the insensitivity of the measure (gross fluorescence) and the number of persons possibly exposed at a county fairs. Both male and female participants were affected, as well as most age and role groups.

This study had some unique characteristics. Digital photography of a fluorescent powder on hands was a successful surrogate for contamination. However, this rather gross measure was likely insensitive when one considers how few bacterial or viral particles are needed to cause certain zoonotic diseases. The black light box was also successful in blinding participants to their contamination status, since they were unable to see inside the box, and few seemed to grasp the experimental nature of the study.

Some of our study findings were unanticipated. We expected contamination proportions to vary by age, gender, and role because we expected these factors to affect the amount of contact with birds and handwashing behavior. However the rates did not vary by these variables. This finding could be due to the study's limited power to detect such differences. If the differences between those exposed and those unexposed were statistically significant (e.g., also occurring in a similar study with a larger sample size), they might be consistent with studies that suggest that animal handlers (exhibitors) practice better hygiene compared to nonhandlers in the same environment. Alternatively, animal handlers may engage in other behavior that affects their contamination status, such as handling enough animals that the surrogate powder wears away more quickly than it would for someone who does not handle animals.

This theoretical model had limitations. Hand contamination with the fluorescent powder was considered a surrogate for pathogen transmission in this study; however, hand contamination of a pathogen does not necessarily lead to transmission. Transmission is dependent upon the amount of inoculated pathogen (dose), the ability of the pathogen to cause disease (virulence), and the ability of the host to defend against infection (host susceptibility) (*17*). These variables are complex and difficult to measure in settings such as a county fair. Additionally, such variables often vary by pathogen and host; hence, we measured only surrogate markers for exposure because such exposure is a requirement for disease to occur.

GloGerm powder contamination may or may not be reflective of true pathogen transmission. The product is useful in handwashing training because it is generally not visible to the naked eye and persons are usually unaware that they have become contaminated. In our study, GloGerm was additionally useful because study participants were also unaware that a chicken was contaminated. Proper handwashing removes the powder, as it would pathogens. However, the amount of time the powder remains on a person's hands without handwashing varies and may be different from the amount of time that a pathogen would be viable on hands. In addition, dusting the chicken with powder is an attempt to model pathogen shedding, but this practice may not truly reflect the amount of pathogens an infected bird would shed. The undersides of the birds were dusted to model fecal shedding and dispersal of pathogens. However, the amount of powder used may be higher or lower than true pathogen shedding.

The study design was further limited in that we did not account for time after exposure when photographs were taken. Since participants could drop by any time of the day, the time after exposure and duration of exposure likely varied between participants. In both the feasibility and pilot studies, the return rate was low, and tracking down participants was difficult. If similar studies are conducted in the future, a reward system might be used to increase compliance.

Petting zoos and agricultural fairs are common in the Midwest and attract many thousands of people. While concern about viral and bacterial zoonotic disease transmission in these settings is growing, they are not usually thought of as a public health concern. The observations from this modest study, even with the limitations described above, suggest that live poultry exhibits may pose a disease transmission risk. Of particular concern is the relatively high proportion of powder transmission to poultry show visitors, who have casual and limited exposure to poultry. Larger future studies of similar design might help identify specific risk factors for zoonotic disease transmission and appropriate interventions for such settings. As a minimum contribution, these study data suggest that hygienic educational programs and disease prevention programs are warranted in poultry exhibits.
